# Taxonomic Characterization and Short-Chain Fatty Acids Production of the Obese Microbiota

**DOI:** 10.3389/fcimb.2021.598093

**Published:** 2021-06-16

**Authors:** M. Carmen Martínez-Cuesta, Rosa del Campo, María Garriga-García, Carmen Peláez, Teresa Requena

**Affiliations:** ^1^ Department of Food Biotechnology and Microbiology, Instituto de Investigación en Ciencias de la Alimentación, CIAL (CSIC-UAM), Madrid, Spain; ^2^ Microbiology Department, Hospital Universitario Ramón y Cajal and Instituto Ramón y Cajal de Investigación Sanitaria (IRYCIS), Madrid, Spain; ^3^ Endocrine Department, Hospital Universitario Ramón y Cajal, Madrid, Spain

**Keywords:** diversity, microbiota, obesity, metabolic activity, short-chain fatty acids, *in vitro* incubations

## Abstract

Intestinal microbiota seems to play a key role in obesity. The impact of the composition and/or functionality of the obesity-associated microbiota have yet to be fully characterized. This work assessed the significance of the taxonomic composition and/or metabolic activity of obese- microbiota by massive 16S rRNA gene sequencing of the fecal microbiome of obese and normoweight individuals. The obese metabolic activity was also assessed by *in vitro* incubation of obese and normoweight microbiotas in nutritive mediums with different energy content. We found that the microbiome richness and diversity of the two groups did not differ significantly, except for Chao1 index, significantly higher in normoweight individuals. At phylum level, neither the abundance of *Firmicutes* or *Bacteroidetes* nor their ratio was associated with the body mass index. Besides, the relative proportions in *Collinsella*, *Clostridium* XIVa, and *Catenibacterium* were significantly enriched in obese participants, while *Alistipes*, *Clostridium sensu stricto*, *Romboutsia*, and *Oscillibacter* were significantly diminished. In regard to metabolic activity, short-chain fatty acids content was significant higher in obese individuals, with acetate being the most abundant followed by propionate and butyrate. Acetate and butyrate production was also higher when incubating obese microbiota in mediums mimicking diets with different energy content; interestingly, a reduced capability of propionate production was associated to the obese microbiome. In spite of the large interindividual variability, the obese phenotype seems to be defined more by the abundance and/or the absence of distinct communities of microorganism rather than by the presence of a specific population.

## Introduction

Obesity is a global health priority and also represents a risk factor in the development of other metabolic diseases, such as diabetes type 2, metabolic syndrome, and cardiovascular diseases. The aetiology of obesity is multifactorial, involving genetic and environmental factors. Among all of these factors, microbiota has been pointed out as a significant player in obesity due to its influence in metabolic and immunological host functions. On one hand, gut microbiota takes part in the energy balance through the production of short chain fatty acids (SCFAs), while on the other hand seems to be involved in the epithelial integrity and regulation of low-grade inflammation associated to obesity.

Initial and further studies showed a large-phylum microbiota shift in obesity with considerable increase of *Firmicutes* in detriments of *Bacteroidetes* (Turnbaugh et al., 2009; Koliada et al., 2017). On the contrary, other reports did not show significant changes in the relative proportions of these two phyla or even others did report inverse findings (Duncan et al., 2008; Tims et al., 2013). These dissimilarities could be explained by differences between studies concerning sample size, anthropometric, and clinical characteristics of individuals and methodologies used for microbiota assessment. Other studies paid attention to unravel which bacterial families, genera, or species may be linked to obesity. Recently, Peters et al. (2018) reported an increase abundance of *Streptococcaceae* and *Lactobacillaceae* and decreased abundance of *Christensenellaceae*, *Clostridiaceae*, and *Dehalobacteriaceae* in the microbiota of obese American adults. To encourage this statement, other studies also found a positive correlation between the abundance of *Christensenellaceae* and a lean phenotype (Goodrich et al., 2014; Oki et al., 2016). Additionally, some studies emphasized a positive correlation among obesity and the *Lactobacillus* population density, particularly *Lactobacillus reuteri*, whereas *Lactobacillus casei*/*paracasei* and *Lactobacillus plantarum* were less represented in the obese microbiome (Million et al., 2012; Million et al., 2013). Meanwhile, *Bifidobacterium* seems to be more abundant in lean individuals. In addition, cross-sectional studies have shown that certain gut bacterial populations, such as *Akkermansia muciniphila*, *Faecalibacterium prausnitzii*, and *Methanobrevibacter smithii* correlated to a lean phenotype and a healthy status (Dao et al., 2016; Derrien et al., 2017). However, the existence of higher levels of *M. smithii* in patients with anorexia nervosa has been also reported (Armougom et al., 2009). Beyond doubt, gut microbiota plays a role in obesity but the relative proportions of the phyla and/or taxonomic-specific changes associated with the phenotype are complex and remain unclear and controversial. Nevertheless, obese microbiota dysbiosis is associated to a lower bacterial diversity, lower gene richness and an alteration of the metabolic functionality (Cotillard et al., 2013).

Further than to community structure fluctuations, differences in the microbiota’s functionality have been associated to obesity. Thus, some *in vitro* and *in vivo* studies have shown higher fecal SCFA concentrations in stool samples of obese individuals than their lean counterparts, due to an increased microbial energy harvest in the obese microbiome (Schwiertz et al., 2010). Also, obese individual seems to produce more colonic SCFAs than lean subjects due to differences in colonic microbiota (Rahat-Rozenbloo et al., 2014). Nevertheless, the interplay between microbiota and the amount and ratio of SCFAs and its role in obesity are quite complex because of the contradictory reports to date.

The question has now become whether we can identify specific members and/or specific functionalities of the gut microbiota that are more relevant than others to the causative role of that microbiota in human obesity and/or we can identify specific functionalities of the gut microbiota in obese individuals. Therefore, the main objective of this work was to characterize the taxonomic composition and functionality of fecal microbiota from obese and normal weight individuals in order to outline the significance of the taxonomic changes in its metabolic activity when subjected to diets with different energy content.

## Materials and Methods

### Volunteers and Sampling

A total of 26 human fecal samples were analyzed in this study. Feces were collected from 13 normoweight (N) (7 female, 6 male; body mass index = 18 - 25 kg m^2^) and 13 obese (O) adult volunteers (7 female, 6 male; body mass index > 30 kg m^2^) attended at the Endocrine Department of the University Hospital Ramón y Cajal (Madrid, Spain). The exclusion criteria for both groups included the use of antibiotics during the preceding 6 months, metabolic and inflammatory diseases, and intake of probiotics and prebiotics. The exclusion criteria also include a history of infectious diseases, cancer, and autoimmune diseases, and the selected volunteers were not related to each other. All volunteers gave written informed consent to the protocol, which had been approved by the Clinical Ethics Committee of Hospital Ramón y Cajal with the code 394/14 and Spanish Council of Scientific Research (CSIC; Spain). Stool samples were collected under aseptic conditions in sterile and screw-top containers and immediately stored at −80°C.

### DNA Isolation

Genomic DNA was extracted from fecal samples (0.1 g) previously thawed at room temperature following the protocol described by Moles et al. (2013). Briefly, after a first centrifugation in sterile saline solution, pellets were suspended in an extraction buffer containing lysozyme and lysostaphin and 3 M sodium acetate; microbial cells were subsequently lysed by mechanical disruption with glass beads (0.1 mm diameter zirconia/silica) (Sigma), using a FastPrep disruptor (QBioGene, Irvine, CA, USA) at a speed setting of 6.0 m/s for 30 s. Extraction was performed with phenol/chloroform/isoamyl alcohol (25:24:1) (Sigma). DNA was precipitated by adding 0.6 volumes of isopropanol, washed with 70% ethanol, allowed to air dry, and finally suspended in DNase, RNase-free water (Sigma-Aldrich). The DNA yield was measured using a NanoDropH ND-1000 UV spectrophotometer (Nano-Drop Technologies).

### 16S rDNA Amplicon-Based Metagenomics

DNA samples were sent to FISABIO (Valencia, Spain) for massive 16S rDNA gene V3–V4 amplicon sequencing on the Ilumina MiqSeq platform and for bioinformatic analyses. The primers were selected from previously described ones (Klindworth et al., 2013). Phylum-, family-, and genus-level taxonomic assignment of sequences that passed quality control were completed using the Ribosomal Database Project classifier software (Wang et al., 2007) within an 80% confidence threshold. Chao1 and Shannon indexes representing species richness and diversity, respectively, were also studied. Bioinformatic analyses were performed with R statistical software (R project, Statistical Software) and several open-source libraries. The quantitative data of the reads were homogenized by using their relative percentages of the total reads of each sample to facilitate the comparison between samples. Finally, the Galaxy Huttenhower Platform (http://huttenhower.sph.harvard.edu/galaxy) was used in order to calculate the Linear Discriminant Effect Size Analysis (LEfSe) algorithm and to obtain cladograms in which microbial taxa that explain significant differences among groups of samples were represented. A free software platform was used according to paper instructions (Segata et al., 2011). The sequencing data were deposited at Digital CSIC (https://digital.csic.es) and is accessible at http://dx.doi.org/10.20350/digitalCSIC/12597.

### *In Vitro* Incubations With Intestinal Microbiota

Colon conditions were simulated in double-jacketed reactors set up at pH 6.5 and 37°C. The reactors were continuously flushed with nitrogen for maintaining anaerobic conditions for the oxygen-sensitive intestinal microbial communities. Reactors were filled with two different nutritive mediums, standard nutritive medium (Barroso et al., 2015) that represents a normal energy (NE) medium and a high-energy (HE) medium (Payne et al., 2012) characterized by a high content of high-glycemic index carbohydrates (digestible starch) and simple carbohydrates (fructose). HE contains 45% more fermentable carbohydrates than the NE medium. Reactors were inoculated with 20% (w/v) of a pool of fecal samples from either normoweight or obese individuals; each pool (N and O) was derived using fecal samples (5 g each) from five individuals; these fecal samples that were previously collected under aseptic conditions, aliquoted and immediately stored at −80°C were thawed, pooled, and homogenized in anaerobic conditions with sodium phosphate buffer (0.1 M, pH 7.0), containing 1 g/L sodium thioglycolate as reducing agent (De Boever et al., 2000). The fecal slurry was aliquoted, snap-frozen in liquid nitrogen and stored at −80°C to enable the same microbiota in replicate experiments. Aliquots of the pooled microbiotas were subsequently incubated with the different media under anaerobic conditions for 24 h at 37°C and pH 6.8. Samples from the reactors were immediately stored at −20°C for SCFAs and ammonium analysis.

### Short-Chain Fatty Acids (SCFAs)

Supernatants from fecal samples and reactor incubations were filtered and 20 µl were further quantified using a Rezex ROA Organic Acids HPLC column (Phenomenex, Macclesfield, UK) at 50°C, with 0.005 M sulphuric acid in HPLC grade water as a mobile phase and at a flow rate of 0.6 ml·min^−1^ (Sanz et al., 2005). HPLC system (Jasco, Tokyo, Japan) equipped with a UV-975 detector and elution profile was monitored at 210 nm. Quantification of the samples was obtained through calibration curves of acetic, propionic, butyric, and lactic acids in concentrations between 0.5 and 100 mM. All samples were analyzed in triplicate.

### Ammonium

The filtered supernatants from fecal samples and reactor incubations describe above were incubated with the Nessler’s reagent for 5 min at room temperature as previously described (Streuli and Averell, 1970). Absorbance was measured using a Varioskan Flash Reader (Thermo Electron Corporation) at 425 nm. For preparation of a standard curve, a dilution series of ammonium chloride was prepared in the range of 0 to 0.2 mM. Samples were analyzed in triplicate.

### Statistical Analysis

Non-normally distributed data were summarized using medians and interquartile ranges (Q1 and Q3). For comparisons between the two groups, the unpaired t-test for parametric data or the Mann-Whitney and Kolmogorov Smirnof tests for non-parametric data were used. In all cases, *P* values of <0.05 were considered to be significant. Statistical analyses were performed using STATISTICA program for Windows, version 7.1.

## Results

### Diversity Analyses

The median number of operational taxonomic units (OTUs) in the 26 samples was 110.262 (ranging from 51.464 to 141.622); lower numbers of reads were associated to obese individuals although without statistical differences. For deciphering the estimated richness of the microbiome and its evenness, Shannon and Chao1 alpha diversity indexes were studied.

**Figure 1:**
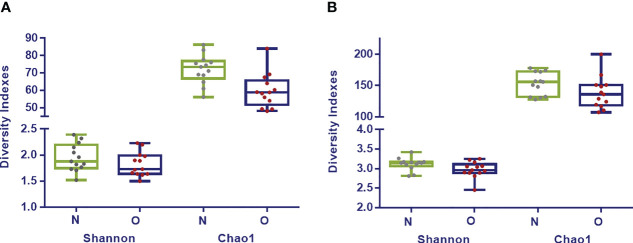
Diversity Indexes Shannon and Chao1 in fecal samples from normoweight (N, 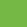) and obese (O, 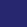) individuals at family **(A)** and genus **(B)** taxonomical levels. The median, minimum, and maximum values are shown.

Shannon index analyzed at phylum (*p*= 0.34), family (*p*= 0.20) and genus (*p*= 0.06) taxonomic levels showed higher values for the normoweight group although no significant differences were found between the two groups. Regarding Chao1, significant differences between the two groups were found at phylum (*p*= 0.008) and family (*p*= 0.002) levels; at deeper taxonomic level, no significant differences were observed (**Figure 1**).

### Taxonomical Assignment of the Bacterial Sequences

**Figure 2:**
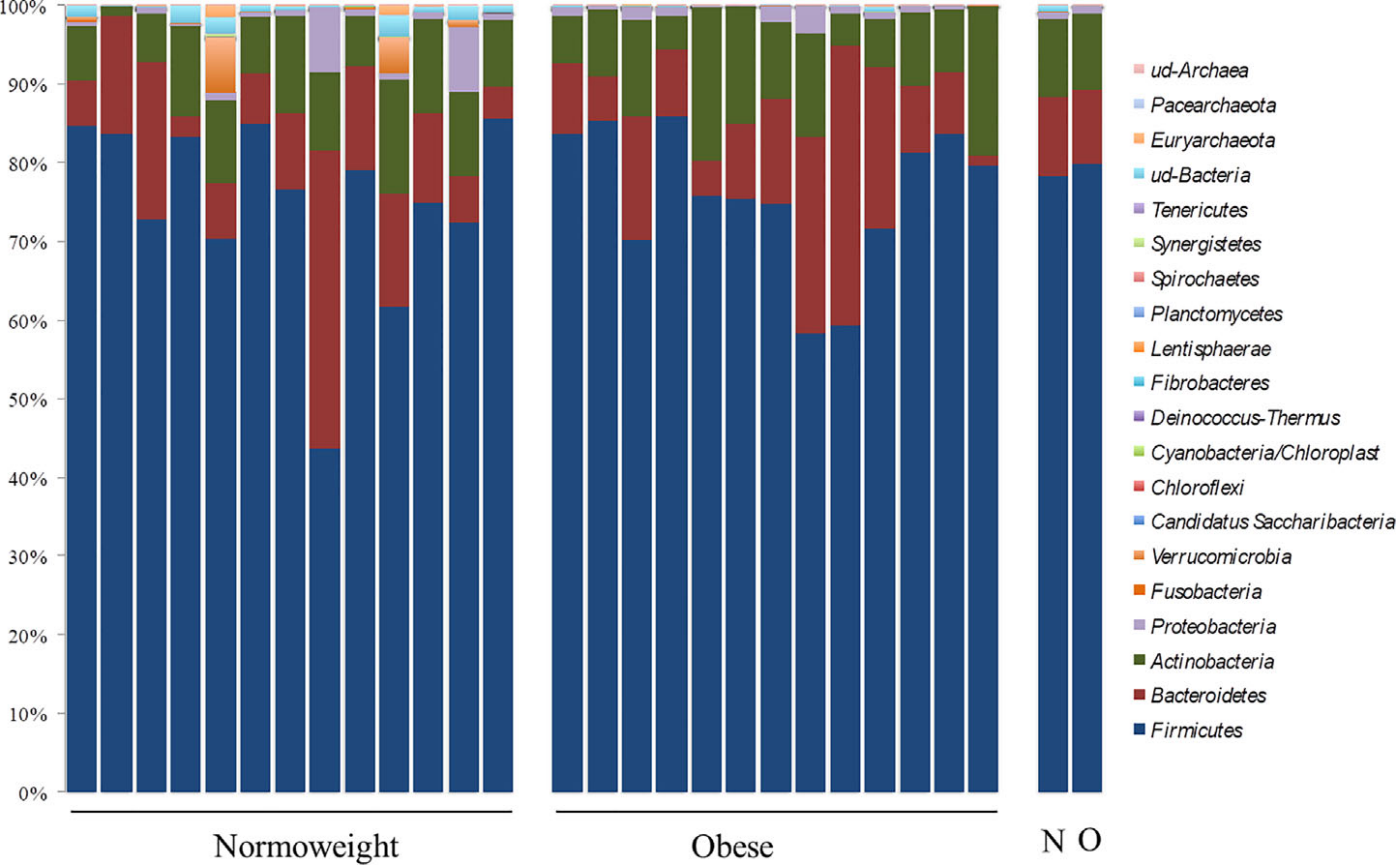
Phylum distribution in the fecal samples analyzed from normoweight (N) and obese (O) individuals. The right columns represent the median value of each group.

Differential abundance of taxa through OTU levels was examined. The most abundant phyla were *Firmicutes* (76.28%; 43.74–85.90%) and *Bacteroidetes* (12.28%; 1.33–37.80%) followed by *Actinobacteria* (9.51%; 0.96–19.34%) and *Proteobacteria* (0.69%; 0.04–8.15%). At this taxonomical level, significant differences between the two groups N and O were only observed for *Verrumicrobia* (*p* < 0.05) (**Figure 2**). OTUs in *Verrucomicrobia* were found in high proportion in two subjects of the N group (4.61% and 7.00%). OTUs corresponding to other phyla were detected but with a relative abundance below 0.05%.

Contrary to several previous reports, abundances of the two most prevalent phyla, *Firmicutes* and *Bacteroidetes*, were not associated with the body mass index (BMI) (*p*= 0.98 and *p*= 0.75, respectively); the *Firmicutes*/*Bacteroidetes* (F/B) ratio was also not associated with the BMI groups because no significant differences were measured among obese and normoweight individuals (*p*= 0.897).

**Figure 3:**
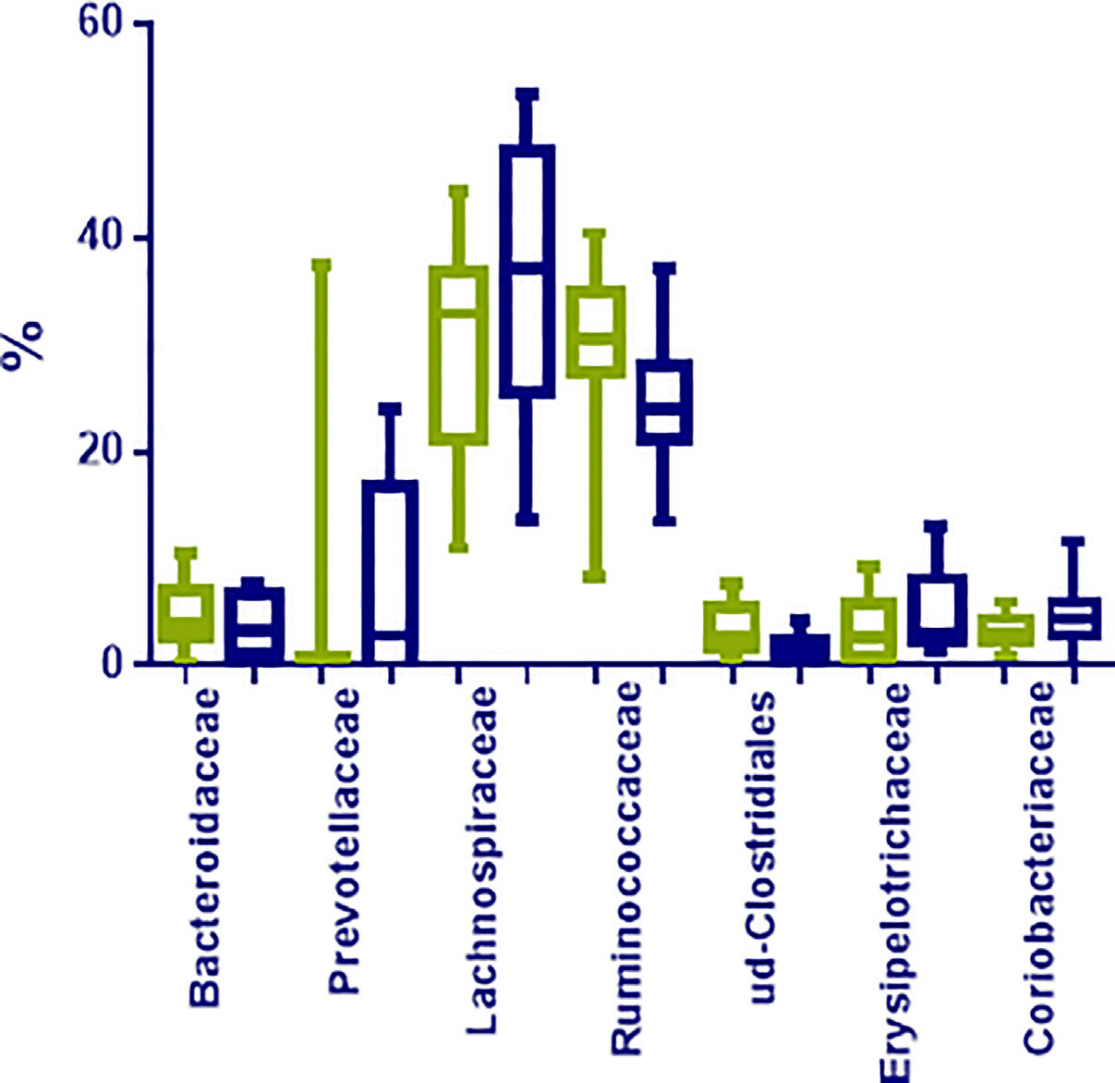
Most represented groups at family level in fecal samples from normoweight (N, 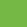) and obese (O, 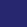) individuals. The median, minimum, and maximum values are shown.

At family level, *Lachnospiraceae* (35.12%; 10.97–53.36%), *Ruminococcaceae* (27.53%; 8.35–70.41%), *Bifidobacteriaceae* (5.91%; 0.01–11.70%), *Coriobacteriaceae* (3.98%; 0.02–11.70%), *Bacteriodaceae* (3.72%; 0.06–10.36%), and *Erysipelotrichaceae* (3.04%; 0.48–13.00%) were the most represented microbial groups; among them, only OTUs in *Ruminococcaceae* were significantly enriched in N compared to O individuals (*p* < 0.05) (**Figure 2**). Additionally, OTUs in *Rikenellaceae* (*p*= 0.03), *Peptostreptococcaceae* (*p*= 0.01), and many unclassified OTUs within *Clostridiales* (*p*= 0.01), were also less represented in obese individuals (**Figure 3**).

**Figure 4:**
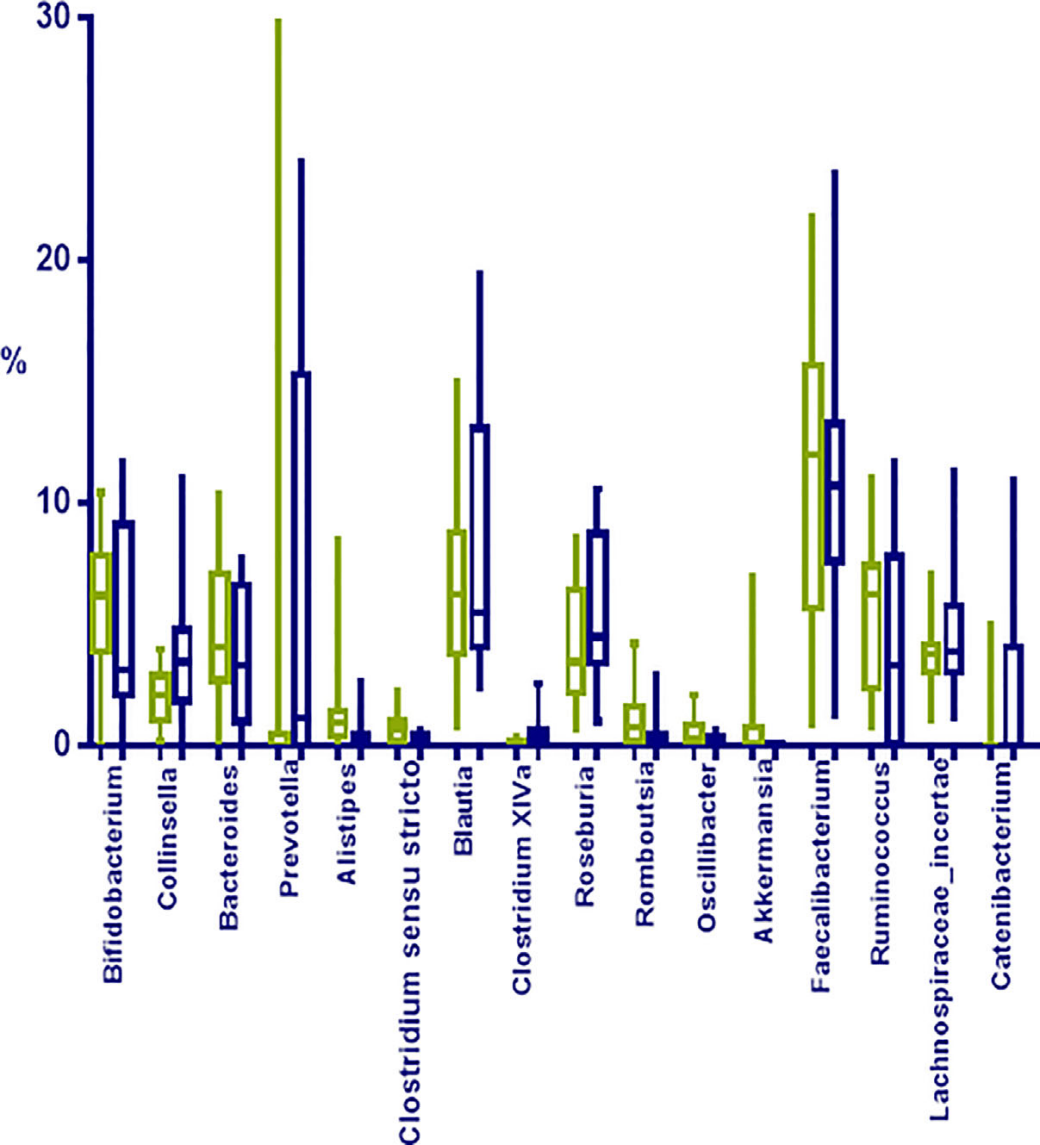
Most represented groups at genera level in fecal samples from normoweight (N, 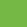) and obese (O, 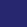) individuals. The median, minimum, and maximum values are shown.

**Figure 5:**
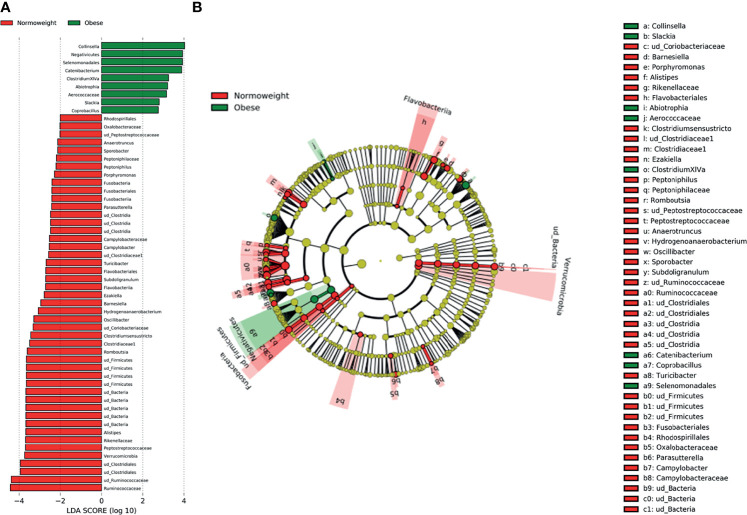
Characterization of microbiomes by LEfSe analysis and LDA. **(A)** Histogram of the LDA scores (log10) computed for features with differential abundance in normal weight (N) and obese (O) subjects. **(B)** Cladograms showing the significant differences of gut microbiota composition in normoweight (N) and obese (O) subjects.

Regarding deep taxonomic level and considering all 26 samples, a total of 323 bacterial genera were detected, although 37 genera represented approximately 90% of the microbiome; being the most abundant *Faecalibacterium* (11.28%; 0.88–3.62%), *Blautia* (5.96%; 0.78–19.40%), *Bifidobacterium* (5.90%; 0.01–11.67%), *Ruminococcus* (5.13%; 0.01–11.75%), *Roseburia* (4.20%; 0.69–10.59%), and *Lachnospiraceae_incertae_sedis* (3.82%; 1.02–11.30%);

When comparing obese to normoweight participants, OTUs in *Collinsella* (*p*=0.04), *Clostridium* XIVa (*p*=0.01) and *Catenibacterium* (*p*= 0.02) were significantly enriched in obese participants while *Alistipes* (*p*=0.03), *Clostridium sensu stricto* (*p*=0.01), *Romboutsia* (*p*=0.02), and *Oscillibacter* (*p*=0.03) were significantly diminished (**Figure 4**). The significant differences in the taxa abundance between O and N individuals were explored by the LEfSE analysis, and the results are represented in **Figure 5**. At the genus level, *Collinsella*, *Negativicutes*, *Selenenomonadales*, *Catenibacterium*, and *Clostridium* XIVa were overrepresented in the obese group (LDA score ≥ 3), while *Alistipes*, *Romboutsia*, *Oscillibacter*, *Clostridium sensu stricto*, and *Hidrogenoanaerobacterium*, were enriched in the normoweight group (LDA score ≥ 3) (**Figure 5A**). Even though the density of various genera significantly differs between both groups, a slight impact of these variations in the global microbiota represented by a cladogram could be observed (**Figure 5B**).

### Microbiota and Fecal Short-Chain Fatty Acids and Ammonium Concentrations

**Figure 6:**
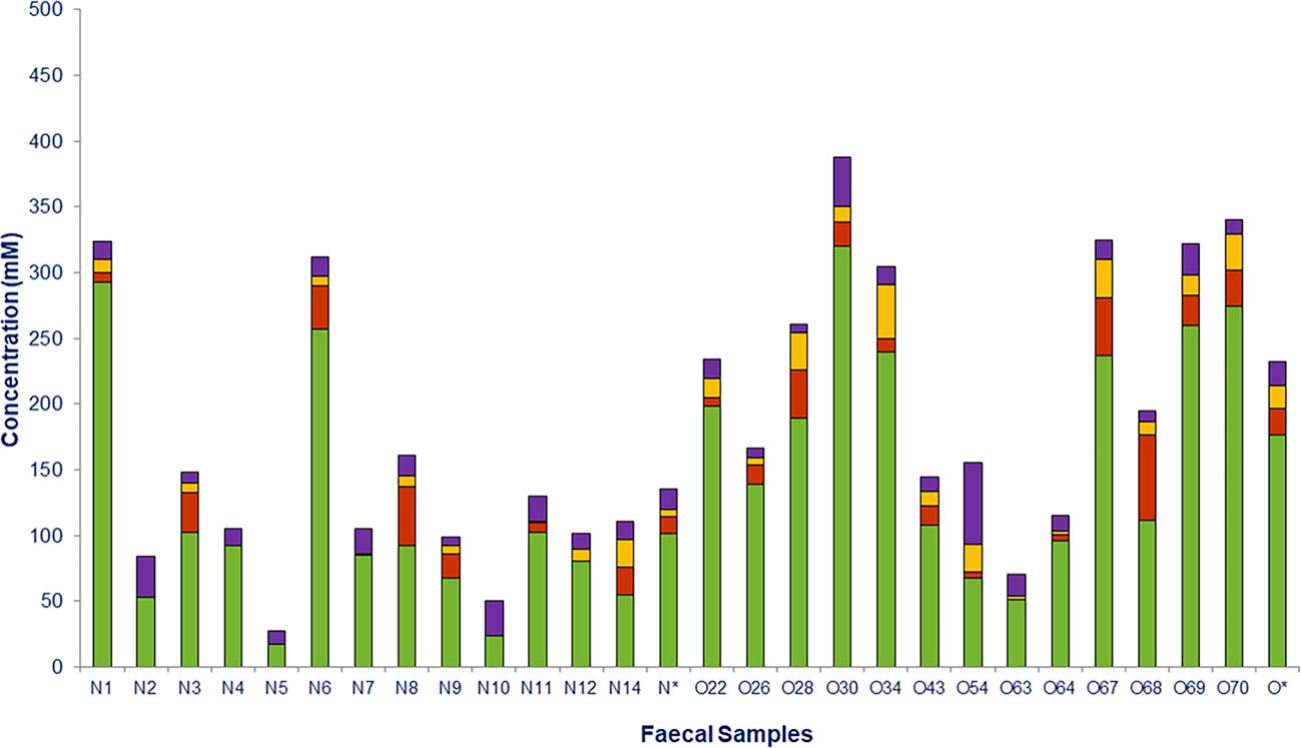
Short chain fatty acids (SCFAs) and lactate measured in fecal samples from normoweight (N) and obese (O) individuals: acetate (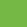), propionate (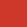), butyrate (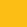), and lactate (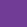). The column (*) represents the median value of each group.

Fecal concentrations of SCFAs and lactate in all individuals are shown in **Figure 6**. Total SCFAs were significantly higher in obese (214.01 ± 27.53 mM) than in normoweight (119.70 ± 24.95 mM) individuals, with the most abundant SCFAs being acetate followed by propionate and butyrate (**Figure 6**). All three SCFAS were detected in obese individuals (except propionate in one of them), whereas butyrate and propionate were not detected in the feces of four and five of the normal weight individuals, respectively. Significant differences were found between the two groups (N and O) for acetate (*p* =0.033) and butyrate (*p* = 0.004).

Pearson’s correlation coefficient using SCFAs and genera taxa as covariates showed a positive correlation (*p* < 0.05) between acetate and *Streptococcus* (0.49), *Coprococcus* (0.46), *Dorea* (0.47), *Roseburia* (0.39) and a negative correlation with *Oscillibacter* (−0.46); butyrate was found to be significantly associated with unclassified OTUs within *Firmicutes* (−0.41) whereas propionate was positively correlated with *Collinsella* (0.57) and *Roseburia* (0.65) and negatively correlated with *Alistipes* (−0.40) and *Oscillibacter* (−0.51).

Regarding the amount of ammonium in feces, higher values were measured in obese (1.02 ± 0.13 mM) compared to normoweight (0.74 ± 0.07 mM) individuals although no significant differences were found between the two groups (*p*= 0.077). Pearson´s coefficient analyses showed that ammonium concentrations were positively correlated (*p* < 0.05) to *Ruminococcus* 2 group (0.71), *Streptococcus* (0.57), and *Bacteroide*s (0.57). In addition acetate and ammonium were also positively correlated (0.61); a negative correlation was found with *Anaerostipes* (−0.40).

**Figure 7:**
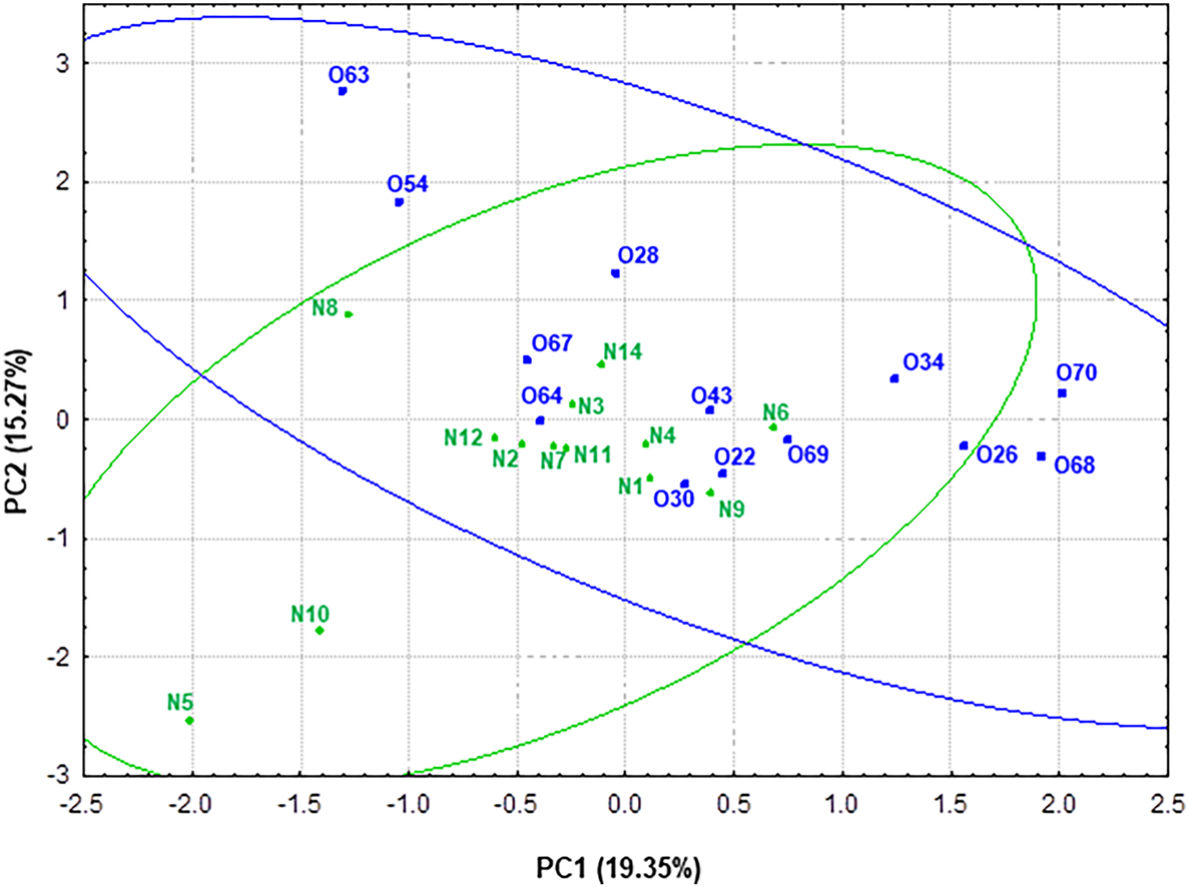
Representation of the samples from normoweight (N) and obese (O) individuals in the plane defined by the two first components (PC1 and PC2) resulting from a PCA that takes into account both genera taxonomic groups and the metabolic activity—SCFAs and ammonium—data.

Principal component analysis (PCA) was performed in order to obtain a simplified view of the changes in metabolic activity -SCFAs, lactate and ammonium- measured in the fecal samples related to gut microbiota. For a better understanding of the data, the scores of the different individuals considering genus taxonomic level (microbiota) and metabolic activity were plotted in the plane delimited by the first two components (**Figure 7**). PC1 explained 19.35% of the variance. This component was positively correlated (loading < 0.70) with taxonomic groups *Blautia* (0.81), *Ruminococcus* 2 (0.81) and *Clostridium* XVIII (0.73). Thus, higher values of PC1 correlated to higher presence of these microbial groups. Interestingly, PC2, which explained 15.27% of the variance, was positively correlated with *Clostridium* XIVa (0.70) and *Catenibacterium* (0.78) (**Supplementary Figure S1**).

### Short-Chain Fatty Acids and Ammonium Production by the N and O Microbiotas in Nutritive Mediums With Different Energy Content

Fecal concentrations of SCFAs and lactate produced by the different microbiotas (N and O) when incubated in mediums with different energy content (NE and HE) are shown in **Table 1**. The highest production of SCFAs was measured in HE medium when incubated with the O microbiota except for propionate. Production of propionate was significantly higher (p < 0.05) in NE medium incubations by N microbiota (4.26 ± 1.62 mM) compared to O microbiota (0.92 ± 0.42 mM); no propionate production was measured in HE medium by the O microbiota. Lactate highest production was detected in HE medium for N microbiota (118.74 ± 21.623 mM).

**Table 1:** Short chain fatty acids and lactate production (mM) during *in vitro* incubations for 24 h of Normoweight (N) and Obese (O) microbiotas in nutritive mediums with different energy content, Normal Energy (NE) and High Energy (HE).

	NE		HE	
	N	O	N	O
Acetate	48.34 ± 3.61	46.85 ± 4.68	44.83 ± 1.88	60.58^*^ ± 4.39
Butyrate	5.77 ± 1.18	11.90^*^ ± 1.64	3.73 ± 0.94	19.39^*^ ± 2.56
Propionate	4.26 ± 1.62	0.92^*^ ± 0.43	0.33 ± 0.13	nd^*^
Lactate	38.30 ± 7.32	14.63 ± 0.79	118.71 ± 21.63	41.10^*^ ± 11.42

Data are expressed as mean ± standard deviation; for a given energy medium, ^*^ denotes significant differences (p<0.05) in the production of SCFAs and lactate between the two microbiotas (N and O); nd, not detected.

Regarding ammonium production during *in vitro* incubations, no significant differences were found in the different mediums for both N and O microbiotas assessing 7.90 ± 0.63 mM and 7.09 ± 0.95 mM, respectively, in NE medium; when incubations were performed in HE medium, ammonium production decreased significantly compared to NE medium, although no significant differences were found between N (3.98 ± 0.15 mM) and O microbiotas (4.71 ± 0.94 mM).

## Discussion

In this study, we compared the taxonomic composition and metabolic activity of the obese and normoweight microbiome towards unravelling the makeup of obese-associated gut microbiota. The microbial profile of fecal samples from normal weight and obese volunteers was approached by massive sequencing of 16S rDNA genes, clustered into operational taxonomic units (OTUs) and taxonomically classified. Beyond that, the potential impact of the taxonomic changes of the obese microbiota in its metabolic activity was assessed by carrying out *in vitro* incubations of these different microbiotas in nutritive mediums with different energy content.

Globally and when analyzing the diversity indexes, we observed that the microbiome richness and diversity was higher for the normal weight (N) group although did not seem to differ significantly; except for the Chao1 index that gives more weight to the low abundance species (Kim et al., 2017), and was significantly higher in the N group. Gut bacterial richness was correlated with obese phenotypes and associated metabolic markers such as adiposity, insulin resistance and dyslipidemia (Rastelli et al., 2018). Though not always the case (Walters et al., 2014; Kasai et al., 2015), most studies of overweight and obese people show a dysbiosis characterized by a lower diversity (Turnbaugh et al., 2009). In regards to this, a low microbiota richness and biodiversity was also reported in obese individuals when compared to normal weight French subjects, whereas this pattern was not found between obese and normal weight Saudis individuals (Yasir et al., 2015). Despite the fact that there is a tremendous variability between individuals, other factors among them such as geographical location and dietary habits have a major influence on the composition, diversity, and metabolic capacity of the gut microbiota.

It is often assumed that a diverse microbiome is associated to a stable and healthy microbiome as a low microbial diversity appears as a common feature in most metabolic, immune and other related diseases (Mosca et al., 2016); however, this assumption cannot be applied in all cases especially as diversity indexes focus on the ecological structure and do not take into account the species composition or the interaction between microbes (Johnson and Burnet, 2016). Nevertheless, it is accepted the more diverse the microbiome, the more adaptable it will be to perturbations (Heiman and Greenway, 2016).

Here, the taxonomic signatures showed neither of the two most abundant prevalent phyla *Firmicutes* and *Bacteroidetes* or the *Firmicutes*/*Bacteroidetes* (F/B) ratio to be associated to BMI. Previously, obesity has been characterized by an altered intestinal F/B ratio associated with a greater relative abundance of *Firmicutes* (Ley et al., 2005). While these results seem to be consistent in rodent animal studies, larger human studies have failed to replicate this signature both at baseline level and after weight loss (Million et al., 2013; Hu et al., 2015). Similarly, Peters et al. (2018) also showed no differences in F/B ratio between obese and lean individuals in a large study conducted in an American adult population. However, a significant increase in relative abundance of *Firmicutes* and higher F/B ratio in overweight and obese compared to normal-weight and lean adults has been reported in a Ukraine population (Koliada et al., 2017). The lack of consistency in the reported results may in part be a reflection of the limitations of the current tools and study designs. In addition, gut microbiota is influenced by many external environmental factors such as nutritional habits, physical exercises and geographical location.

Our data suggest that the obese phenotype is characterized more by the abundance and/or absence of distinct communities of microorganism rather than by the presence of a specific population. In fact we identified at family level several OTUS such as *Ruminococcaceae*, *Rikenellacea*e, *Peptostreptococcaceae* and many unclassified OTUs within *Clostridiales* to be less represented in the obese microbiome. Menni et al. (2017) also pointed out the possibility that *Ruminococcaceae* may be functionally linked to a lean phenotype but further functional studies are needed to assess if this is the case. While Lv et al. (2019) also found a negative relationship between *Rikenellaceae* and BMI, the taxa *Peptostreptococcaceae* has been reported elsewhere to be associated with obesity and intestinal inflammation in previous studies (Monk et al., 2016), as much as three-fold higher in obese when compared to normal weight children and adolescents (Nirmalkar et al., 2018).

In terms of genera, different groups were enriched in the obese microbiomes, among them *Clostridium* XIVa and *Collinsella*. *Clostridium* cluster XIVa members belong to the *Lachnospiraceae* family and many representatives of this cluster are efficient producers of butyrate which is associated to energy metabolism and an energy source for gut epithelial cells. As seen here, an increased abundance of this cluster in diet-induced obese rodents (Jiao et al., 2018) and a decrease during a dietary intervention inducing weight loss was observed previously (Remely et al., 2015). A relative abundance of *Collinsella*, a genus belonging to *Actinobacteria* has been associated to obesity and insulin resistance (Frost et al., 2019); *Collinsella* was also enriched in the microbiome of obese adolescents and significantly decreased in abundance during weight loss program (Nirmalkar et al., 2018; Frost et al., 2019).

Apart from taxonomic composition, there is growing evidence from both human and animal studies that suggests a link between gut microbiome, SCFAs and obesity development (Zhao, 2013). Fecal SCFAs are typically measured to reflect the colonic production of the SCFAs. However, several other factors such as colonic SCFA absorption, colonic transit time, differences in dietary intake and intestinal microbiota also contribute. SCFAs appear to have a complex and pleiotropic role in obesity; on one hand, SCFAs may enhance energy harvest and contribute to excess lipogenesis in the liver, but also concurrently reduce inflammation, sensitize tissues to insulin, contribute to satiety and improve gut barrier function (Nehra et al., 2016). The results here presented are consistent with other studies that have also reported higher SCFAs concentration in the feces of overweight and obese individuals when compared to counterpart lean volunteers (Schwiertz et al., 2010; Rahat-Rozenbloo et al., 2014), suggesting overall that increased fecal concentrations of SCFAs are associated with obesity. However, other reports have confirmed that administration of exogenous acetate, propionate, or butyrate prevent weight gain in diet induced mice and overweight humans (Lin et al., 2012; Chambers et al., 2015); besides, a high fiber intake has been linked to a rise in health-promoting SCFAs production (Rios-Covián et al., 2017) along with a reduction of detrimental compounds, such as indole and hydrogen sulphide (Zhao et al., 2018).

In addition, obese microbiome seems to be more efficient in harvesting energy from the diet, through the production of SCFAs, than lean microbiome (Turnbaugh et al., 2008; Fernandes et al., 2014); this pattern emerged in the microbiota of obese children showing a higher ability to ferment carbohydrates when compared to lean volunteers (Goffredo et al., 2016). In the results shown here, higher concentrations of acetate and butyrate were measured when incubating the obese microbiota in HE medium. Obese microbiome has been previously shown to be enriched in metabolic pathways leading to butyrate and acetate production, the major end products of fermentation (Turnbaugh et al., 2006). On the other hand, propionate production was lower in the *in vitro* incubations involving the O microbiota, even no propionate production was measured in HE medium incubated with the O microbiota; Interestingly, propionate metabolism has not being associated with obesity.

Higher amount of ammonium was measured in obese feces compared to normoweight individuals, although not significantly different did positively correlate with acetate levels. Fecal ammonia excretion was shown to be positively and strongly related to excretion of acetate, propionate, butyrate, and total SCFAs. Low pH values could also explain this as ammonia is freely diffusible and absorbed by colonocytes in the unprotonated form; ammonium ion is not absorbed (McOrist et al., 2011). When assessing ammonium production during incubation in nutritive mediums with different energy content, no differences between the two microbiotas (N and O) were found; nevertheless a significant reduction in ammonium production was measured in HE medium. Previous results in our lab showed that the increase of easily digestible carbohydrates maintains the fermentative functionality of the intestinal microbiota and decreases the proteolytic activity in colonic reactors (Barroso et al., 2016). Some other studies suggest that obese individuals could exhibit disruption of nitrogen disposal, in particular urea cycle function and glutamine formation (Alemany, 2012) and/or an alteration in ammonia detoxification processes (Cho et al., 2017).

Here our results suggest that although there is support for a relationship between the microbial communities found in human feces and obesity, this association seems to be relatively weak. The small sample size used in this study is a limitation, and further studies need to be conducted on a larger population of obese and normal weight individuals to give us a clearer picture of the correlations that exist between the parameters studied here. Other features, such as the large interindividual variation in the microbiome’s structure, and environmental factors, such as diet should also account for this. Alternatively, it is possible that taxonomic signature of obesity relies on finer species (OTU) levels, rather than at phylum, family and genus level.

Obesity is a complex disease and it has a multifactorial etiology; the gut microbiota participates in a complex interaction with the host metabolism; and the gut microbes, directly and indirectly interplay with all the organs *via* specific metabolites, hormones, and neurotransmitters. Microbiota dysbiosis is thought to promote obesity through different mechanisms such an improved capacity for energy harvest and storage, gut permeability and inflammation, nervous and endocrine routes, among others. In regard to metabolic activity studied here, SCFAs content is higher in fecal obese samples; besides, this trend is also observed when assessing acetate and butyrate production of the obese microbiota under diets with different energy contents; interestingly, a reduced capability of propionate production is associated to the obese microbiome. Most likely, the differences could become clearer when unravelling the functionality of the microbiome and the metabolites produced from the different taxonomic groups. A metabolomics approach can provide key information to clarifying the possible role of the microbiota in obesity. Thus, to get a better picture of the relationship between obesity and microbiota, functional microbiomic approaches need to be employed.

## Data Availability Statement

The original contributions presented in the study are publicly available. The Sequencing Data was deposited at Digital CSIC (https://digital.csic.es) and is accessible at http://dx.doi.org/10.20350/digitalCSIC/12597.

## Ethics Statement

The studies involving human participants were reviewed and approved by Clinical Ethics Committee of Hospital Ramón y Cajal with the code 394/14 and Spanish Council of Scientific Research (CSIC; Spain). The patients/participants provided their written informed consent to participate in this study.

## Author Contributions

MM-C, CP, and TR conceived and supervised the study. All authors participated in the methodology. MM-C, RC, CP, and TR interpreted the results. MM-C wrote the first draft of the originally manuscript. MM-C, RC, CP, and TR provided a critical revision of the manuscript. MM-C, CP, and TR provided the funding project. All authors contributed to the article and approved the submitted version.

## Funding

This work was supported by the Spanish Ministry of Science and Innovation (AGL2016-75951-R and PID2019-106071RB-I00 Grants).

## Conflict of Interest

The authors declare that the research was conducted in the absence of any commercial or financial relationships that could be construed as a potential conflict of interest.

## Supplementary Material

Supplementary Figure 1 Projection of the variables genera taxa, SCFAs and ammonium on the factor-plane defined by the two principal components PC1 and PC2.

## References

[B1] Alemany M. (2012). The Problem of Nitrogen Disposal in the Obese. Nutr. Res. Rev. 25, 18–28. doi: 10.1017/S0954422411000163 22309896

[B2] Armougom F. Bittar F. Stremler N. Rolain J. M. Robert C. Dubus J. C.. (2009). Microbial Diversity in the Sputum of a Cystic Fibrosis Patient Studied With 16S rDNA Pyrosequencing. Eur. J. Clin. Microbiol. Infect. Dis. 28, 1151–1154. doi: 10.1007/s10096-009-0749-x 19449045

[B3] Barroso E. Cueva C. Peláez C. Martínez-Cuesta M. C. Requena T. (2015). Development of Human Colonic Microbiota in the Computer-Controlled Dynamic Simulator of the Gastrointestinal Tract SIMGI. LWT - Food Sci. Technol. 61, 283–289. doi: 10.1016/j.lwt.2014.12.014

[B4] Barroso E. Montilla A. Corzo N. Peláez C. Martínez-Cuesta M. C. Requena T. (2016). Effect of Lactulose-Derived Oligosaccharides on Intestinal Microbiota During the Shift Between Media With Different Energy Contents. Food Res. Int. 89, 302–308. doi: 10.1016/j.foodres.2016.08.025 28460919

[B5] Chambers E. S. Viardot A. Psichas A. Morrison D. J. Murphy K. G. Zac-Varghese S. E.. (2015). Effects of Targeted Delivery of Propionate to the Human Colon on Appetite Regulation, Body Weight Maintenance and Adiposity in Overweight Adults. Gut 64, 1744–1754. doi: 10.1136/gutjnl-2014-307913 25500202 PMC4680171

[B6] Cho K. Moon J. S. Kang J. H. Jang H. B. Lee H. J. Park S. I. (2017). Combined Untargeted and Targeted Metabolomic Profiling Reveals Urinary Biomarkers for Discriminating Obese From Normal-Weight Adolescents. Pediatr. Obes. 12, 93–101. doi: 10.1111/ijpo.12114 26910390

[B7] Cotillard A. Kennedy S. P. Kong L. C. Prifti E. Pons N. Le Chatelier E.. (2013). Dietary Intervention Impact on Gut Microbial Gene Richness. Nature 500, 585–588. doi: 10.1038/nature12480 23985875

[B8] Dao M. C. Everard A. Aron-Wisnewsky J. Sokolovska N. Prifti E. Verger E. O.. (2016). *Akkermansia Muciniphila* and Improved Metabolic Health During a Dietary Intervention in Obesity: Relationship With Gut Microbiome Richness and Ecology. Gut 65, 426–436. doi: 10.1136/gutjnl-2014-308778 26100928

[B9] De Boever P. Deplancke B. Verstraete W. (2000). Fermentation by Gut Microbiota Cultured in a Simulator of the Human Intestinal Microbial Ecosystem is Improved by Supplementing a Soygerm Powder. J. Nutr. 130, 2599–2606. doi: 10.1093/jn/130.10.2599 11015496

[B10] Derrien M. Belzer C. de Vos W. M. (2017). Akkermansia Muciniphila and Its Role in Regulating Host Functions. Microb. Pathog. 106, 171–181. doi: 10.1016/j.micpath.2016.02.005 26875998

[B11] Duncan S. H. Lobley G. E. Holtrop G. Ince J. Johnstone A. M. Louis P.. (2008). Human Colonic Microbiota Associated With Diet, Obesity and Weight Loss. Int. J. Obes. (Lond.) 32, 1720–1724. doi: 10.1038/ijo.2008.155 18779823

[B12] Fernandes J. Su W. Rahat-Rozenbloom S. Wolever T. M. Comelli E. M. (2014). Adiposity, Gut Microbiota and Faecal Short Chain Fatty Acids Are Linked in Adult Humans. Nutr. Diabetes 4, e121. doi: 10.1038/nutd.2014.23 24979150 PMC4079931

[B13] Frost F. Storck L. J. Kacprowski T. Gärtner S. Rühlemann M. Bang C.. (2019). A Structured Weight Loss Program Increases Gut Microbiota Phylogenetic Diversity and Reduces Levels of *Collinsella* in Obese Type 2 Diabetics: A Pilot Study. PloS One 14, e0219489. doi: 10.1371/journal.pone.0219489 31318902 PMC6638920

[B14] Goffredo M. Mass K. Parks E. J. Wagner D. A. McClure E. A. Graf J.. (2016). Role of Gut Microbiota and Short Chain Fatty Acids in Modulating Energy Harvest and Fat Partitioning in Youth. J. Clin. Endocrinol. Metab. 101, 4367–4376. doi: 10.1210/jc.2016-1797 27648960 PMC5095239

[B15] Goodrich J. K. Waters J. L. Poole A. C. Sutter J. L. Koren O. Blekhman R.. (2014). Human Genetics Shape the Gut Microbiome. Cell 159, 789–799. doi: 10.1016/j.cell.2014.09.053 25417156 PMC4255478

[B16] Heiman M. L. Greenway F. L. (2016). A Healthy Gastrointestinal Microbiome Is Dependent of Dietary Diversity. Mol. Metab. 5, 317–320. doi: 10.1016/j.molmet.2016.02.005 27110483 PMC4837298

[B17] Hu H. J. Park S. G. Jang H. B. Choi M. K. Park K. H. Kang J. H.. (2015). Obesity Alters the Microbial Community Profile in Korean Adolescents. PloS One 10, e0134333. doi: 10.1371/journal.pone.0134333 26230509 PMC4521691

[B18] Jiao N. Baker S. S. Nugent C. A. Tsompana M. Cai L. Wang Y.. (2018). Gut Microbiome may Contribute to Insulin Resistance and Systemic Inflammation in Obese Rodents: A Meta-Analysis. Physiol. Genomics 50, 244–254. doi: 10.1152/physiolgenomics.00114.2017 29373083

[B19] Johnson K. V. Burnet P. W. (2016). Microbiome: Should We Diversify From Diversity? Gut Microbes 7, 455–458. doi: 10.1080/19490976.2016.1241933 27723427 PMC5103657

[B20] Kasai C. Sugimoto K. Moritani I. Tanaka J. Oya Y. Inoue H.. (2015). Comparison of the Gut Microbiota Composition Between Obese and Non-Obese Individuals in a Japanese Population, as Analyzed by Terminal Restriction Fragment Length Polymorphism and Next-Generation Sequencing. BMC Gastroenterol. 15, 100. doi: 10.1186/s12876-015-0330-2 26261039 PMC4531509

[B21] Kim B. R. Shin J. Guevarra R. Lee J. H. Kim D. W. Seol K. H.. (2017). Deciphering Diversity Indices for a Better Understanding of Microbial Communities. J. Microbiol. Biotechnol. 27, 2089–2093. doi: 10.4014/jmb.1709.09027 29032640

[B22] Klindworth A. Pruesse E. Schweer T. Peplies J. Quast C. Horn M.. (2013). Evaluation of General 16S Ribosomal RNA Gene PCR Primers for Classical and Next-Generation Sequencing-Based Diversity Studies. Nucleic Acids Res. 41, e1. doi: 10.1093/nar/gks808 22933715 PMC3592464

[B23] Koliada A. Syzenko G. Moseiko V. Budovska L. Puchkov K. Perederiy V.. (2017). Association Between Body Mass Index and Firmicutes/Bacteroidetes Ratio in an Adult Ukrainian Population. BMC Microbiol. 17, 120. doi: 10.1186/s12866-017-1027-1 28532414 PMC5440985

[B24] Ley R. E. Backhed F. Turnbaugh P. Lozupone C. A. Knight R. D. Gordon J. I. (2005). Obesity Alters Gut Microbial Ecology. Proc. Natl. Acad. Sci. U. S. A. 102, 11070–11075. doi: 10.1073/pnas.0504978102 16033867 PMC1176910

[B25] Lin H. V. Frassetto A. Kowalik E. J. Jr Nawrocki A. R. Lu M. M. Kosinski J. R.. (2012). Butyrate and Propionate Protect Against Diet-Induced Obesity and Regulate Gut Hormones *Via* Free Fatty Acid Receptor 3-Independent Mechanisms. PloS One 7, e35240. doi: 10.1371/journal.pone.0035240 22506074 PMC3323649

[B26] Lv Y. Qin X. Jia H. Chen S. Sun W. Wang X. (2019). The Association Between Gut Microbiota Composition and Body Mass Index in Chinese Male College Students, as Analyzed by Next-Generation Sequencing. Br. J. Nutr. 9, 1–17. doi: 10.1017/S0007114519001909 31397240

[B27] McOrist A. L. Miller R. B. Bird A. R. Keogh J. B. Noakes M. Topping D. L.. (2011). Fecal Butyrate Levels Vary Widely Among Individuals But Are Usually Increased by a Diet High in Resistant Starch. J. Nutr. 141, 883–889. doi: 10.3945/jn.110.128504 21430242

[B28] Menni C. Jackson M. A. Pallister T. Steves C. J. Spector T. D. Valdes A. M. (2017). Gut Microbiome Diversity and High-Fibre Intake Are Related to Lower Long-Term Weight Gain. Int. J. Obes. 41, 1099–1105. doi: 10.1038/ijo.2017.66 PMC5500185 28286339

[B29] Million M. Angelakis E. Maraninchi M. Henry M. Giorgi R. Valero R.. (2013). Correlation Between Body Mass Index and Gut Concentrations of *Lactobacillus Reuteri*, *Bifidobacterium Animalis*, *Methanobrevibacter Smithii* and *Escherichia Coli* . Int. J. Obes. 37, 1460–1466. doi: 10.1038/ijo.2013.20 PMC3826031 23459324

[B30] Million M. Angelakis E. Paul M. Armougom F. Leibovici L. Raoult D. (2012). Comparative Meta-Analysis of the Effect of *Lactobacillus* Species on Weight Gain in Humans and Animals. Microb. Pathog. 53, 100–108. doi: 10.1016/j.micpath.2012.05.007 22634320

[B31] Moles L. Gómez M. Heilig H. Bustos G. Fuentes S. de Vos W.. (2013). Bacterial Diversity in Meconium of Preterm Neonates and Evolution of Their Fecal Microbiota During the First Month of Life. PloS One 8, e66986. doi: 10.1371/journal.pone.0066986 23840569 PMC3695978

[B32] Monk J. M. Lepp D. Zhang C. P. Wu W. Zarepoor L. Lu J. T.. (2016). Diets Enriched With Cranberry Beans Alter the Microbiota and Mitigate Colitis Severity and Associated Inflammation. J. Nutr. Biochem. 28, 129–139. doi: 10.1016/j.jnutbio.2015.10.014 26878790

[B33] Mosca A. Leclerc M. Hugot J. P. (2016). Gut Microbiota Diversity and Human Diseases: Should We Reintroduce Key Predators in Our Ecosystem? Front. Microbiol. 7, 455. doi: 10.3389/fmicb.2016.00455 27065999 PMC4815357

[B34] Nehra V. Allen J. M. Mailing L. J. Kashyap P. C. Woods J. A. (2016). Gut Microbiota: Modulation of Host Physiology in Obesity. Physiol 31, 327–335. doi: 10.1152/physiol.00005.2016 PMC5005265 27511459

[B35] Nirmalkar K. Murugesan S. Pizano-Zárate M. L. Villalobos-Flores L. E. García-González C. Morales-Hernández R. M.. (2018). Gut Microbiota and Endothelial Dysfunction Markers in Obese Mexican Children and Adolescents. Nutrients 10 (12), 2009. doi: 10.3390/nu10122009 PMC6315777 30572569

[B36] Oki K. Toyama M. Banno T. Chonan O. Benno Y. Watanabe K. (2016). Comprehensive Analysis of the Fecal Microbiota of Healthy Japanese Adults Reveals a New Bacterial Lineage Associated With a Phenotype Characterized by a High Frequency of Bowel Movements and a Lean Body Type. BMC Microbiol. 16, 284. doi: 10.1186/s12866-016-0898-x 27894251 PMC5127096

[B37] Payne A. N. Chassard C. Lacroix C. (2012). Gut Microbial Adaptation to Dietary Consumption of Fructose, Artificial Sweeteners and Sugar Alcohols: Implications for Host-Microbe Interactions Contributing to Obesity. Obes. Rev. 13, 799–809. doi: 10.1111/j.1467-789X.2012.01009.x 22686435

[B38] Peters B. A. Shapiro J. A. Church T. R. Miller G. Trinh-Shevrin C. Yuen E.. (2018). A Taxonomic Signature of Obesity in a Large Study of American Adults. Sci. Rep. 8, 9749. doi: 10.1038/s41598-018-28126-1 29950689 PMC6021409

[B39] Rahat-Rozenbloo S. Fernandes J. Gloor G. B. Wolever T. M. (2014). Evidence for Greater Production of Colonic Short-Chain Fatty Acids in Overweight Than Lean Humans. Int. J. Obes. 38, 1525–1531. doi: 10.1038/ijo.2014.46 PMC3970979 24642959

[B40] Rastelli M. Knauf C. Cani P. D. (2018). Gut Microbes and Health: A Focus on the Mechanisms Linking Microbes, Obesity, and Related Disorders. Obes. (Silver Spring) 26, 792–800. doi: 10.1002/oby.22175 PMC5947576 29687645

[B41] Remely M. Tesar I. Hippe B. Gnauer S. Rust P. Haslberger A. G. (2015). Gut Microbiota Composition Correlates With Changes in Body Fat Content Due to Weight Loss. Benef. Microbes 6, 431–439. doi: 10.3920/BM2014.0104 25609655

[B42] Rios-Covián D. Salazar N. Gueimonde M. de Los Reyes-Gavilan C. G. (2017). Shaping the Metabolism of Intestinal Bacteroides Population Through Diet to Improve Human Health. Front. Microbiol. 8, 376. doi: 10.3389/fmicb.2017.00376 28326076 PMC5339271

[B43] Sanz M. L. Polemis N. Morales V. Corzo N. Drakoularakou A. Gibson G. R.. (2005). *In Vitro* Investigation Into the Potential Prebiotic Activity of Honey Oligosaccharides. J. Agric. Food Chem. 53, 2914–2921. doi: 10.1021/jf0500684 15826039

[B44] Schwiertz A. Taras D. Schäfer K. L. Beijer S. Bos N. A. Donus C.. (2010). Microbiota and SCFA in Lean and Overweight Healthy Subjects. Obesity 18, 190–195. doi: 10.1038/oby.2009.167 19498350

[B45] Segata N. Izard J. Waldron L. Gevers D. Miropolsky L. Garrett W. S.. (2011). Metagenomic Biomarker Discovery and Explanation. Genome Biol. 12, R60. doi: 10.1186/gb-2011-12-6-r60 21702898 PMC3218848

[B46] Streuli C. A. Averell P. R. (1970). “The Analytical Chemistry of Nitrogen and Its Compounds”, in Chemical Analysis, vol. 28. Eds. Elving P. J. Kolthoff I. M. (New York: Wiley-Interscience), 52–62.

[B47] Tims S. Derom C. Jonkers D. M. Vlietinck R. Saris W. H. Kleerebezem M.. (2013). Microbiota Conservation and BMI Signatures in Adult Monozygotic Twins. ISME J. 7, 707–717. doi: 10.1038/ismej.2012.146 23190729 PMC3603393

[B48] Turnbaugh P. J. Bäckhed F. Fulton L. Gordon J. I. (2008). Diet-Induced Obesity is Linked to Marked But Reversible Alterations in the Mouse Distal Gut Microbiome. Cell Host Microbe 3, 213–223. doi: 10.1016/j.chom.2008.02.015 18407065 PMC3687783

[B49] Turnbaugh P. J. Hamady M. Yatsunenko T. Cantarel B. L. Duncan A. Ley R. E.. (2009). A Core Gut Microbiome in Obese and Lean Twins. Nature 457, 480–484. doi: 10.1038/nature07540 19043404 PMC2677729

[B50] Turnbaugh P. J. Ley R. E. Mahowald M. A. Mardis E. R. Gordon J. I. (2006). An Obesity-Associated Gut Microbiome With Increased Capacity for Energy Harvest. Nature 444, 1027–1031. doi: 10.1038/nature05414 17183312

[B51] Walters W. A. Xu Z. Knight R. (2014). Meta-Analyses of Human Gut Microbes Associated With Obesity and IBD. FEBS Lett. 588, 4223–4233. doi: 10.1016/j.febslet.2014.09.039 25307765 PMC5050012

[B52] Wang Q. Garrity G. M. Tiedje J. M. Cole J. R. (2007). Naive Bayesian Classifier for Rapid Assignment of rRNA Sequences Into the New Bacterial Taxonomy. Appl. Environ. Microbiol. 73, 5261–5267. doi: 10.1128/AEM.00062-07 17586664 PMC1950982

[B53] Yasir M. Angelakis E. Bibi F. Azhar E. I. Bachar D. Lagier J. C.. (2015). Comparison of the Gut Microbiota of People in France and Saudi Arabia. Nutr. Diabetes 5, e153. doi: 10.1038/nutd.2015.3 25915742 PMC4423199

[B54] Zhao L. (2013). The Gut Microbiota and Obesity: From Correlation to Causality. Nat. Rev. Microbiol. 11, 639–647. doi: 10.1038/nrmicro3089 23912213

[B55] Zhao L. Zhang F. Ding X. Wu G. Lam Y. Y. Wang X.. (2018). Gut Bacteria Selectively Promoted by Dietary Fibers Alleviate Type 2 Diabetes. Science 359, 1151–1156. doi: 10.1126/science.aao5774 29590046

