# Erratum: Taxonomic Characterization and Short-Chain Fatty Acids Production of the Obese Microbiota

**DOI:** 10.3389/fcimb.2021.781260

**Published:** 2021-10-12

**Authors:** Frontiers Production Office

**Affiliations:** Frontiers Media SA, Lausanne, Switzerland

**Keywords:** diversity, microbiota, obesity, metabolic activity, short-chain fatty acids, *in vitro* incubations

Due to a production error, there was a mistake in **Figures 5**, **6**, and **7** as published. **Figure 5** should contain parts A and B, rather than just part A. What was published as **Figure** 6 should be **Figure** 5B. What was published as **Figure 7** should be **Figure 6**. **Figure 7** was subsequently left out of the article. The corrected **Figures 5**, **6**, and **7** appear below.

**Figure 5:**
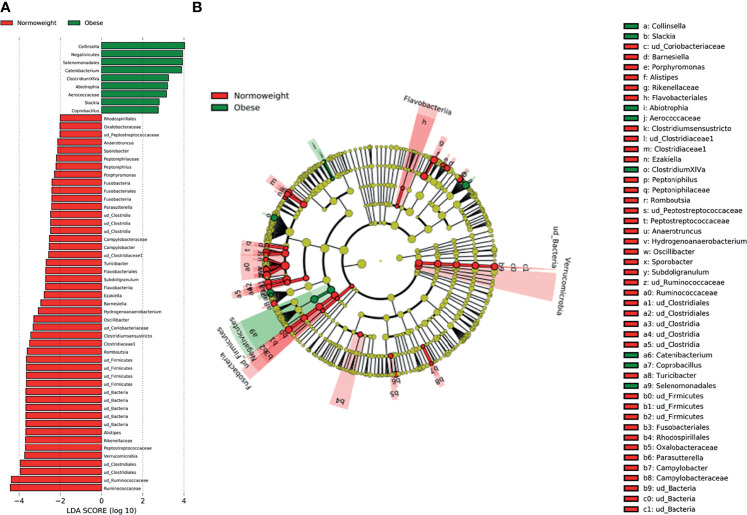
Characterization of microbiomes by LEfSe analysis and LDA. **(A)** Histogram of the LDA scores (log10) computed for features with differential abundance in normal weight (N) and obese (O) subjects. **(B)** Cladograms showing the significant differences of gut microbiota composition in normoweight (N) and obese (O) subjects.

**Figure 6:**
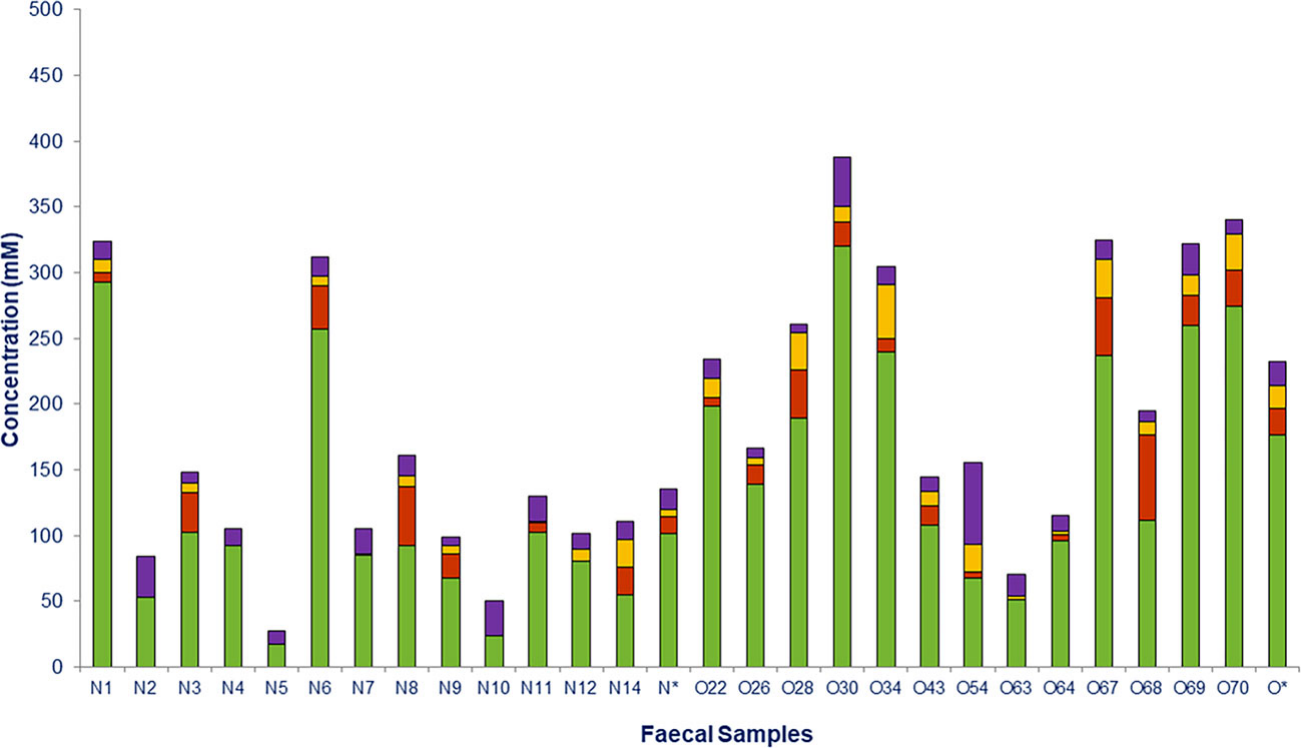
Short chain fatty acids (SCFAs) and lactate measured in fecal samples from normoweight (N) and obese (O) individuals: acetate (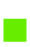), propionate (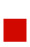), butyrate (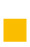), and lactate (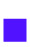). The column (*) represents the median value of each group.

**Figure 7:**
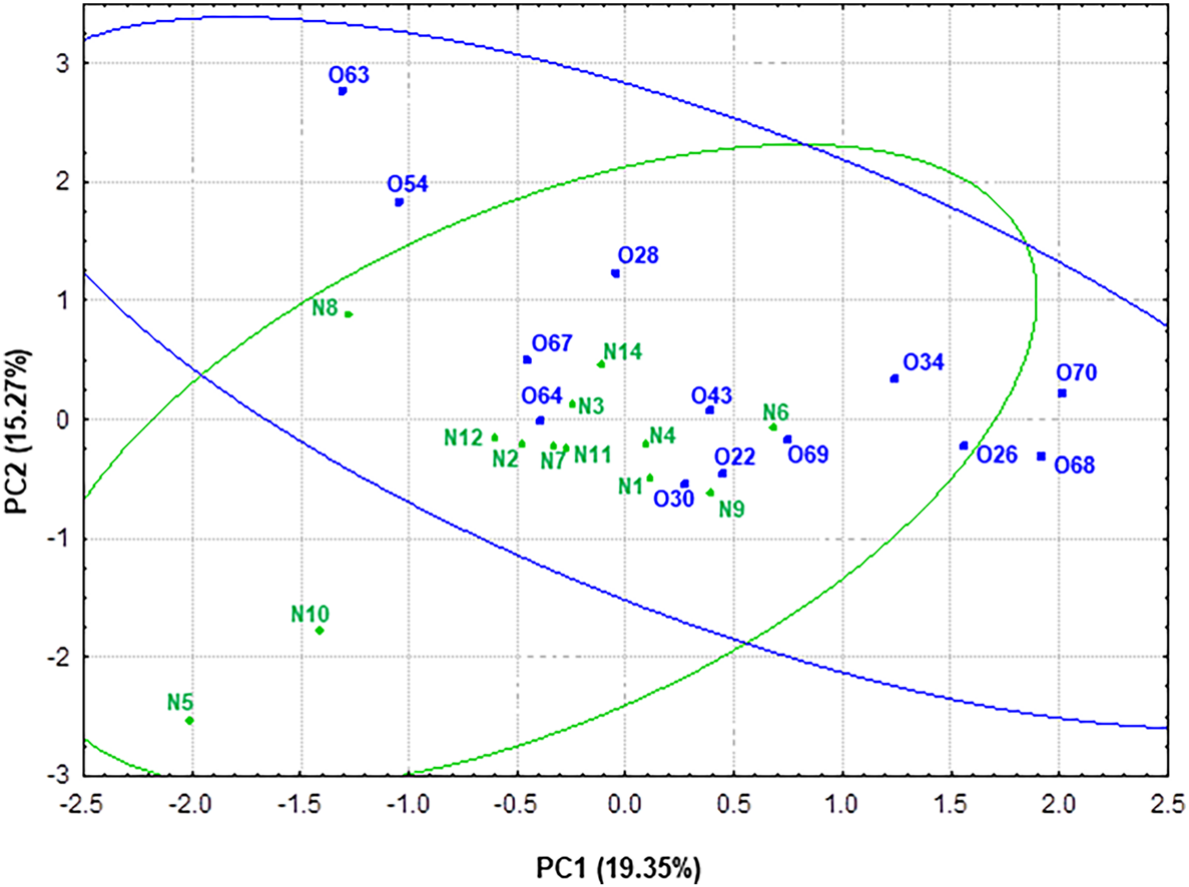
Representation of the samples from normoweight (N) and obese (O) individuals in the plane defined by the two first components (PC1 and PC2) resulting from a PCA that takes into account both genera taxonomic groups and the metabolic activity-SCFAs and ammonium-data.

The publisher apologizes for this mistake.

The original version of this article has been updated.

