# Supplementary figures and images for: Taxonomic Characterization and Short-Chain Fatty Acids Production of the Obese Microbiota
Source: Front Cell Infect Microbiol. 2021 Jun 16;11:598093. doi: 10.3389/fcimb.2021.598093 (PMC8242951; doi:10.3389/fcimb.2021.598093)

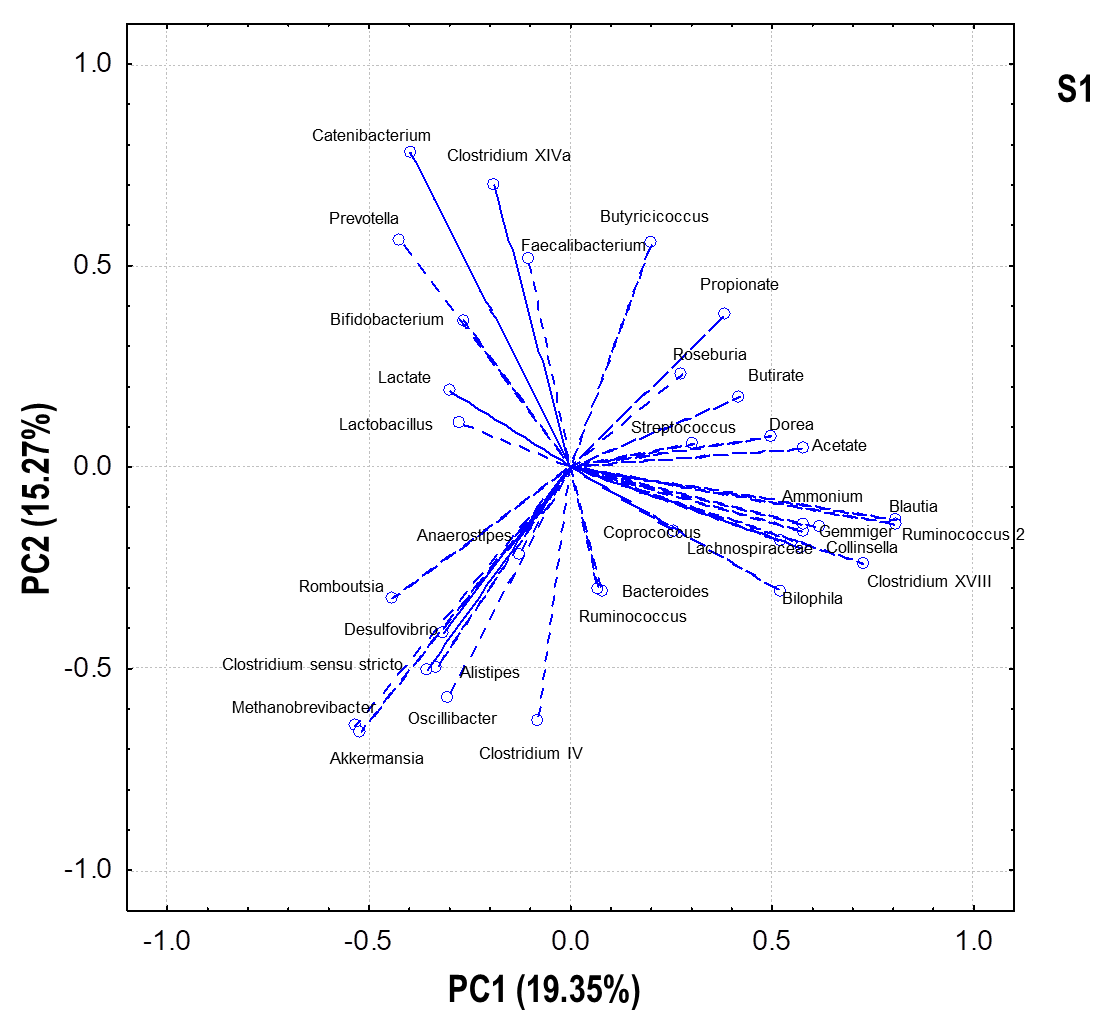

Supplement: Supplementary Figure 1 — Projection of the variables genera taxa, SCFAs and ammonium on the factor-plane defined by the two principal components PC1 and PC2. [file Image_1.tif]
